# ChIP-chip data for identifying target genes and consensus binding sequences of mutant p53 in MDA-MB-468 breast cancer cells

**DOI:** 10.1016/j.dib.2023.109499

**Published:** 2023-08-18

**Authors:** Mai Nhu Uyen Le, Yichong Ning, Jianlin Zhou

**Affiliations:** a State Key Laboratory of Developmental Biology of Freshwater Fish & Key Laboratory of Protein Chemistry and Developmental Biology of the Ministry of Education, College of Life Science, Hunan Normal University, Changsha, Hunan 410081, China; b Chongzuo Key Laboratory of Biomedical Clinical Transformation, The People's Hospital of Chongzuo, Youjiang Medical University for Nationalities, Chongzuo, Guangxi, China

**Keywords:** TP53, Chromatin immunoprecipitation (ChIP), DNA microarray, Transcription factor, DNA binding motif

## Abstract

The tumor suppressor p53 exerts its role mainly as a transcription factor. The TP53 gene, which encodes the p53 protein, is the most commonly mutated gene in human cancers, particularly triple negative breast cancer (TNBC). Variations in the TP53 gene occur mainly in exons 5–8 and result in missense mutations in the DNA-binding domain of the p53 protein that alter DNA binding specificity. To identify the target genes of mutant p53, we performed chromatin immunoprecipitation followed by DNA microarray (ChIP-chip). Briefly, the TNBC cell line MDA-MB-468 containing the endogenous p53-R273H mutation (the arginine residue at position 273 is mutated to a histidine) was cross-linked with 1% formaldehyde and ultrasonically sheared to generate chromatin fragments in a range of 200∼1000 bp. An aliquot of the sheared chromatin was kept as input, and the other chromatin was precipitated with a p53 monoclonal antibody. DNA was purified from the precipitated chromatin and the unprecipitated chromatin (i.e., input), amplified, and labeled with Cy5 (ChIP DNA) or Cy3 (input DNA). Cy5- and Cy3-labeled DNA samples were cohybridized with the NimbleGen Human ChIP-chip 2.1 M Deluxe Promoter Array. The raw and analyzed data are described in this article. They are useful for identifying target genes and consensus binding motifs of the p53 R273H mutant and for further clarifying the molecular mechanism underlying the oncogenic activity of the p53 mutant.

Specifications Table

**Table:** 

Subject	Cancer research
Specific subject area	Chromatin immunoprecipitation, DNA microarray, mutant p53
Data format	Raw, Analyzed
Type of data	Table, Chart
Data collection	MDA-MB-468 cells were cross-linked, ultrasonically sheared, and immunoprecipitated with an anti-p53 antibody. DNA was purified from precipitated chromatin and unprecipitated chromatin (i.e., input), amplified, and labeled with Cy5 (ChIP) or Cy3 (input). The Cy5- and Cy3-labeled DNA samples were cohybridized with the NimbleGen Human ChIP-chip 2.1 M Deluxe Promoter Arrays. After washing, the microarrays were scanned using an Axon 4000B microarray scanner with GenePix 6.0 software. Raw data were extracted as pair files using NimbleGen software (v2.5).
Data source location	College of Life Science, Hunan Normal University, 36 Lushan Road, Changsha, 410081, Hunan, China
Data accessibility	Repository name: Gene Expression Omnibus (GEO) [1] Data identification number: GSE128304 Direct URL to data: https://www.ncbi.nlm.nih.gov/geo/query/acc.cgi?acc=GSE128304

## 1 Value of the Data

• Scientists can use these data to determine the genes whose transcription is regulated by the p53-R273H mutant in MDA-MB-468 cells.
• By analyzing the data, researchers can identify the consensus binding sequence of the p53-R273H mutant using a de novo motif recognition tool.
• The data are useful to further elucidate the pathway by which the p53 mutant plays an oncogenic role.

## 2 Objective

The tumor suppressor p53 is an essential transcription factor involved in the development and progression of various cancers, including breast cancer [2]. Nearly half of all human cancers have a TP53 gene mutation, most of which are missense mutations in the DNA-binding domain of the p53 protein [3,4]. The mutation in the DNA-binding domain impairs its DNA-binding activity and/or alters its DNA-binding specificity [5]. To identify the DNA-binding targets of mutant p53, we performed chromatin immunoprecipitation followed by DNA microarray (ChIP-chip). The triple-negative breast cancer (TNBC) cell line MDA-MB-468 was used for ChIP experiments because it has an endogenous p53-R273H mutation (arginine to histidine mutation at position 273) [6], while the R273H mutation is a missense mutation in the DNA-binding domain and is the third most common mutation in p53 in human cancers [7]. The raw and processed data described in this article are useful for researchers to identify the genes transcriptionally regulated by p53-R273H and to further elucidate the oncogenic mechanism of the p53 mutant.

## 3 Data Description

The dataset presented in this article includes data from triplicate DNA microarray assays (Table 1). ChIP experiments were performed in triplicate. The ChIP-enriched DNA and the input DNA of each ChIP experiment (sample name: GSM3670994, GSM3670995 and GSM3670996) were subjected to microarray assays. NimbleGen Human ChIP-chip 2.1 M Deluxe Promoter Arrays (HG18 Deluxe promoter HX1 arrays) (Roche NimbleGen, Madison, WI, USA) covering the promoter regions (from -7250 bp to + 3250 bp of transcription start sites) of all known human genes were used for the microarray experiments. Microarray assay data (Table 1) were deposited in the NCBI GEO repository (GSE128304). The raw data (*.pair files) contain the signal intensities extracted from the array image. Normalized data (*_ratio.gff files) contain log2 ratio (ChIP/input) values for individual probes normalized with median-centering, quantile normalization, and linear smoothing. Peaks data (*_peaks.gff files) contain all identified peaks representing regions of significant positive enrichment. The normalized data and peaks data can be visualized using SignalMap software (Roche NimbleGen). In addition, the analysis results of the microarray assay data were also deposited in the NCBI GEO repository (GSE128304), including all identified peaks overlapping the promoter region of each transcript (-7250bp ∼ + 3250bp) and a summary list of peaks overlapping the promoter region of the transcripts for the 3 samples. Triplicate ChIP-chip experiments identified 10718,12771, and 12546 genes whose promoters were bound by p53-R273H. A total of 7405 genes overlapped in these three experiments.

**Table 1:** Raw data, normalized data, and peaks for three replicate microarrays.

Sample	Raw data	Normalized data	Peaks
GSM3670994	Sample1_532.pair Sample1_635.pair	Sample1_635_ratio.gff	Sample1_635_ratio_peaks.gff
GSM3670995	Sample2_532.pair Sample2_635.pair	Sample2_635_ratio.gff	Sample2_635_ratio_peaks.gff
GSM3670996	Sample3_532.pair Sample3_635.pair	Sample3_635_ratio.gff	Sample3_635_ratio_peaks.gff

## 4 Experimental Design, Materials and Methods

### 4.1 Cell Culture

MDA-MB-468 cells were obtained from the American Tissue Culture Collection (Manassas, VA, USA) and grown in Leibovitz's L-15 medium supplemented with glutamine, antibiotics and 10% fetal bovine serum at 37 °C and 5% CO_2_.

### 4.2 ChIP Assay

MDA-MB-468 cells were cross-linked with 1% formaldehyde for 10 min at room temperature. Glycine (final concentration: 125 mmol/L) was then added and incubated for 10 min to quench the formaldehyde. After the cells were rinsed twice with phosphate buffered saline (PBS), they were collected by centrifugation. The cell pellet was dissolved in nucleolysis buffer (1% SDS, 10 mmol/L EDTA, 50 mmol/L Tris–HCl, pH 8.0) for 10 min and sonicated with a Bioruptor sonicator (Diagenode, Seraing, Belgium) with high power model for 20 cycles (sonication cycle: 30 s ON, 30 s OFF) at 4 °C. Until agarose gel electrophoresis showed that DNA fragments were 200∼1000 bp long, sonicated chromatin was diluted in dilution buffer (0.01% SDS, 1.1% Triton X-100, 1.2 mmol/L EDTA, 167 mmol/L NaCl, 16.7 mmol/L Tris–HCl, pH 8.0) and precipitated with anti-p53 antibody (catalog number 17-613, Millipore, Billerica, MA, USA) and protein A/G magnetic beads (catalog number 16-661, Millipore). An aliquot of the sonicated chromatin was kept as input before precipitation. The precipitated and unprecipitated complexes (i.e., input) were reversibly cross-linked and treated overnight at 65 °C with proteinase K (Fermentas, Burlington, Ontario, Canada). DNA fragments were purified using Qiagen MinElute columns (Qiagen, Hilden, Germany). The ChIP experiment was repeated three times.

### 4.3 DNA Microarray

DNA was amplified using the Complete Whole Genome Amplification Kit (Sigma-Aldrich, St. Louis, MO, USA), purified using the QIAquick PCR Purification Kit (Qiagen), and quantified using a Nanodrop ND -1000 (ThermoFisher, Carlsbad, CA, USA). ChIP-enriched DNA and input DNA were labeled with Cy5 (ChIP) and Cy3 (input), respectively, using the NimbleGen Dual-Color DNA Labeling Kit. The Cy5- and Cy3-labeled DNA samples were cohybridized with the NimbleGen Human ChIP-chip 2.1 M Deluxe Promoter Arrays at 42 °C for 18 h. After hybridization, the microarrays were washed with Nimblegen wash buffer and scanned using an Axon GenePix 4000B microarray scanner with GenePix 6.0 software (Axon, Scottsdale, AZ, USA). Raw data were extracted as pair files using NimbleScan software.

### 4.4 Data Processing

MA plot, a plot of the distribution of the ratio between red and green intensity (M) plotted by the average intensity (A), was used to assess the quality of the raw data. Median centering, quantile normalization, and linear smoothing were performed to normalize the raw data using the Bioconductor packages Ringo [8], limma [9], and MEDME [10] (https://www.bioconductor.org/). After normalization, a normalized log2 ratio (ChIP/input) was generated for each sample and used for peak finding analysis. To evaluate the effects of normalization, a boxplot was created, showing that all arrays were centered at the same level at zero and the distributions of log2 ratios were nearly the same on all slides after normalization. Based on the normalized log2-ratio data (*_ratio.gff files), a permutation-based peak-finding algorithm of NimbleScan v2.5 (Roche-NimbleGen) was applied to find peaks representing significant positive enrichment. NimbleScan detects peaks by searching for 4 or more probes whose signals are above certain cutoff values, ranging from 90% to 15%, using a 500 bp sliding window. The cutoff values are a percentage of a hypothetical maximum equal to the mean + 6 [standard deviation]. Ratio data were randomized 20 times to determine the probability of “false positives”. Each peak was then assigned a false discovery rate (FDR) value based on the randomization. Peaks with an FDR ≤ 0.05 were assigned to genomic features: Transcripts.

## Ethics Statements

The authors have read and follow the ethical requirements for publication in Data in Brief and confirm that the current work does not involve human subjects, animal experiments, or any data collected from social media platforms.

## CRediT authorship contribution statement

**Mai Nhu Uyen Le:** Investigation, Visualization. **Yichong Ning:** Formal analysis, Writing – original draft. **Jianlin Zhou:** Conceptualization, Funding acquisition, Writing – review & editing, Funding acquisition.

## Data Availability

Genome-wide identification of mutant p53 targets (Original data) (Gene Expression Omnibus).
